# Prevalence and risk factors of alcohol and substance abuse among motorcycle drivers in Fars province, Iran

**Published:** 2019-08

**Authors:** Yaser Sarikhani, Seyed Taghi Heydari, Mehrdad Vossoughi, Arash Mani, Zahra Ghoohestani, Kamran Bagheri Lankarani

**Affiliations:** ^*a*^Student Research Committee, Shiraz University of Medical Sciences, Shiraz, Iran.; ^*b*^Health Policy Research Center, School of Medicine, Shiraz University of Medical Sciences, Shiraz, Iran.; ^*c*^Department of Dental Public Health, Oral and Dental Disease Research Center, School of Dentistry, Shiraz University of Medical Sciences, Shiraz, Iran.; ^*d*^Research Center for Psychiatry and Behavior Sciences and Community Based Psychiatric Care Research Center, Shiraz University of Medical Sciences, Shiraz, Iran.; ^*e*^Health Policy Research Center, School of Medicine, Shiraz University of Medical Sciences, Shiraz, Iran.

## Abstract

**Background:**

Motorcycle accidents are the most common cause of injuries, accounting for 49.1% of all trauma cases each year worldwide. Different risk factors for incidence of motorcycle accidents have been identified. Two main factors that increase the risk of traffic accidents among motorcyclists are alcohol and substance abuse. The aim of this present study is to investigate the prevalence of alcohol and substance abuse and its relationship with other risky driving behaviors among motorcycle drivers in Fars province.

**Methods:**

This is a cross sectional study which is performed at Fars province of Iran in 2017. Data from motorcycle drivers were collected using a standard questionnaire in three major cities of Fars province including Shiraz, Jahrom, and Darab at different times of the day. The data includes consumption of alcohol and other substances two hours before driving and some of the risky behaviors during driving.

**Results:**

A total of 1195 drivers with a mean ± SD age of (28.3 ± 8.56) years participated in the study. The prevalence of alcohol, opium, and amphetamines consumption among participants was 13.2%, 4.6% and 1.3% respectively. Prevalence of alcohol or substance consumption two hours before driving was 17.5%. Consumption of alcohol or substance two hours before driving was significantly associated with risky driving behaviors such as using mobile phone during driving, maneuvering while driving, history of driving fines in the past year, and driving over the speed limit (P <0.001). It was also associated with carelessness about safety such as driving with technical defects, not wearing a crash helmet, and history of accident in the past year (P <0.008).

**Conclusions:**

It seems that alcohol and substance abuse has association with other risky driving behaviors among motorcycle drivers. Thus, it is suggested to screen motorcycle drivers for substance and alcohol abuse in order to identify motorcycle drivers at a higher risk of road traffic accidents more efficiently while taking the cost-effectiveness of any ASA assessment program into consideration.

**Keywords:**

Alcohol drinking, Substance-related disorders, Motorcycle, Iran

